# *In silico* analysis of hypo-upregulated genes as biomarkers in lung adenocarcinoma

**DOI:** 10.6026/973206300212128

**Published:** 2025-07-31

**Authors:** Binisha Robinson, Luke Elizabeth Hanna, Shanmughavel Piramanayagam

**Affiliations:** 1 Department of Bioinformatics, Bharathiar University, Coimbatore, Tamil Nadu, India; 2 Department of Virology and biotechnology, ICMR-National Institute for Research in Tuberculosis, Chennai-600031, India

**Keywords:** Lung adenocarcinoma, DNA methylation, gene- expression, hypo-upregulation, biomarker, multi-omics

## Abstract

Lung adenocarcinoma (LUAD), the most common kind of lung cancer, is characterized by altered gene expression and DNA methylation. This study used TCGA to evaluate methylation and expression data from LUAD. Differential analysis revealed 7313 methylated genes and 250 upregulated genes. Integration identified "Hypo-Up" genes, which are hypomethylated and elevated, indicating carcinogenic potential. Further protein-protein interaction studies will reveal important seed genes. Data shows severe epigenetic and transcriptome abnormalities in LUAD and suggest new biomarker possibilities.

## Background:

Lung adenocarcinoma (LUAD) is the most prevalent subtype of non-small cell lung cancer (NSCLC), accounting for roughly 40% of all diagnosed cases [1]. LUAD is a complex disease characterized by aberrant gene expression and DNA methylation patterns. These molecular alterations contribute to uncontrolled cell proliferation, invasion and metastasis, ultimately leading to poor patient prognosis [2]. Delving into the interplay between DNA methylation and gene expression profiles holds immense potential for elucidating the underlying mechanisms of LUAD development and progression [3]. Analyzing gene expression profiles alongside methylation data offers a comprehensive perspective on how these molecular changes influence LUAD biology. By integrating DNA methylation and gene expression data, researchers can identify genes that are hyper methylated (silenced) and under expressed, potentially uncovering novel tumor suppressor genes in LUAD [4]. A number of genes that control the invasion and survival of cancer cells are expressed during this process. Therefore, as prospective pharmacological targets in the drug development process, medications that alter the genes or proteins that control cancer cell survival, metastasis, apoptosis and invasion are crucial [5]. Furthermore, integrating these profiles with clinical data like patient prognosis and response to therapy can lead to the identification of methylation and gene expression biomarkers that can be used for early diagnosis, treatment stratification, and the development of targeted therapies for LUAD patients [6]. Chronic exposure to cigarette smoke induces various genetic and epigenetic alterations, paving the way for tumor initiation and progression [7]. DNA methylation often involves hypermethylation, where a methyl group (CH_3_) is added to specific DNA regions rich in cytosine-guanine dinucleotides (CpG islands). This disrupts gene expression, particularly silencing tumor suppressor genes [8]. Genes crucial for cell cycle control, DNA repair and apoptosis (cell death) are frequently silenced by hyper methylation in LUAD [9, 10]. The transcriptional silence of tumor suppressor genes is frequently linked to CpG island hypermethylation, which is defined by the excessive methylation of promoter-associated CpG islands [11].

The molecular heterogeneity and clonal development seen in lung adenocarcinoma are a result of the dynamic interaction between global hypomethylation and CpG island hypermethylation. Notably, new research has shown many DNA methylation subgroups in lung cancer, each with its own methylation signatures and clinical characteristics [12]. Hypomethylation can lead to the abnormal activation of oncogenes - genes that promote uncontrolled cell division and survival, a hallmark of cancer [13]. Additionally, it might cause genomic instability, increase the risk of mutations and further promoting tumorigenesis. Finding DNA methylation biomarkers has great potential to enhance lung cancer patients' early detection, prognosis and treatment stratification [14]. Gene expression profiling has also revealed significant alterations in LUAD compared to normal lung tissue. The upregulation of oncogenes, genes that promote cell proliferation and survival, is a hallmark of LUAD. Conversely, tumor suppressor genes are often downregulated, contributing to uncontrolled cell growth and tumor formation [15]. This surge in oncogenic activity throws the natural cellular brakes off, leading to a relentless drive for LUAD cells to multiply. Understanding how these upregulated oncogenes function is essential, as they hold the potential to be future targets for therapeutic strategies. By developing drugs that specifically target and inhibit these hyperactive oncogenes, researchers aim to restore cellular balance and halt the out-of- control growth of LUAD tumor [16]. By integrating DNA methylation and gene expression data, researchers can gain a more comprehensive understanding of the interplay between these epigenetic modifications in LUAD. And this approach can shed light on the functional consequences of methylation events and identify genes that are critically involved in lung cancer development [17]. Researchers can identify genes with differential methylation patterns linked to aberrant gene expression in lung cancer by using statistical modeling and computer techniques [18]. Also numerous genes and pathways influenced by changes in DNA methylation have been discovered by these integrative analyses, providing insight into new biomarkers and potential therapeutic targets for precision medicine therapies [19]. Therefore, it is of interest to systematically investigate and report the integrative analysis of DNA methylation and gene expression profiles in lung adenocarcinoma, with the goal of identifying novel biomarkers and potential therapeutic targets relevant to disease diagnosis, prognosis, and treatment.

## Methodology:

## Differentially methylated biomarker gene identification:

## Methylation data collection:

DNA methylation data for this study was retrieved from The Cancer Genome Atlas (TCGA) database, a comprehensive resource for cancer genomics data. The data specifically focused on lung adenocarcinoma (LUAD) and included samples from Asian individuals. Eight samples of normal lung tissue and eight LUAD samples were obtained from the TCGA database using GDC Tool. Illumina Infinium Human Methylation technology was employed by TCGA to generate methylation profiles for these samples. This approach allows for genome-wide analysis of DNA methylation patterns in both normal and cancerous lung tissue from Asian individuals.

## Data pre-processing:

In preparation for analysis, row containing any missing values (NA) were systematically removed from the datasets. Methylation beta values were transformed into M values using the formula m = log2 (beta / (1 - beta)), ensuring a uniform representation across the methylation dataset. Subsequently, the datasets were merged, yielding a consolidated matrix file containing data from eight normal lung tissue samples and eight Lung Adenocarcinoma (LUAD) samples, facilitating comprehensive methylation analysis.

## Identification of DMRs:

To identify regions of the genome exhibiting significant methylation differences between lung adenocarcinoma (LUAD) and normal lung tissue samples, a differential methylation analysis was performed using the R package limma. Limma compares methylation beta values between groups and employs linear models to account for potential confounding factors. To control for the high number of statistical tests inherent in such analyses, p-values were adjusted for false discovery rate (FDR) using the Benjamini and Hochberg (BH) method. A stringent cutoff of adjusted p-value <0.05 was applied to ensure statistically significant differences in methylation. Additionally, differentially methylated CpG sites (DMSs) were required to exhibit an absolute delta beta value greater than 0.2. This threshold defines the minimum magnitude of methylation difference (beta values) considered biologically relevant for identifying DMRs. By combining these criteria, we aimed to identify a robust set of DMRs that are statistically significant and exhibit substantial methylation alterations associated with LUAD development.

## Identification of differentially methylated genes (DMGs):

Following the identification of differentially methylated regions (DMRs), we further explored the association between these regions and genes. DMRs can encompass regulatory elements that influence gene expression. Hypermethylated DMRs, characterized by an adjusted p-value < 0.05 and a delta beta value greater than 0.1, were hypothesized to potentially silence genes located within or nearby the region. Conversely, hypomethylated DMRs, identified with a similar p-value threshold but a delta beta value less than -0.1. Might be associated with increased gene expression. By linking DMRs to genes, we aimed to pinpoint genes whose expression might be regulated by DNA methylation changes and potentially play a role in lung adenocarcinoma development. This analysis allows us to investigate the functional consequences of the identified methylation alteration.

## Gene expression biomarker identification:

## Expression data collection:

DNA methylation data for this study was retrieved from The Cancer Genome Atlas (TCGA) database, a comprehensive resource for cancer genomics data. The data specifically focused on lung adenocarcinoma (LUAD) and included samples from Asian individuals. Eight samples of normal lung tissue and eight LUAD samples were obtained from the TCGA database. Illumina Infinium Human Methylation technology was employed by TCGA to generate methylation profiles for these samples. This approach allows for genome-wide analysis of DNA methylation patterns in both normal and cancerous lung tissue from Asian individuals.

## Pre-processing:

In the pre-processing stage, rows containing zero values were eliminated from the dataset, and the remaining values were rounded to whole numbers for further analysis. Protein-coding genes were selectively retained in the expression profile data during the pre-process.

## Identification of differentially expressed genes (DEGs):

Differential expression between LUAD (N = 5) and normal lung tisssue (N = 2) samples was analyzed with the R limma package. We adjusted each p-value as false discovery rate (FDR) using the Benjamini and Hochberg (BH) method. We used the log-transformed expression value to identify differentially expressed genes (DEGs), including upregulated genes with an adjusted p-value < 0.05 and logFC (fold change) > 1, and downregulated genes with an adjusted p-value < 0.05 and logFC < -1 in LUAD compared with normal lung tisssue.

## Integration of DEG and DMG:

To correlate the relationship between methylation and expression, we analysed differentially methylated and expressed genes (DMEGs). One specific group of interest within DMEGs are genes classified as Hypo-Up. These genes exhibit hypomethylation, meaning a decrease in methylation compared to the normal state. This decrease in methylation is associated with increased expression, suggesting the genes are potentially more active due to the reduced suppressive effect of methylation.

## The protein-protein interaction network analysis:

For protein-protein interaction (PPI) analysis, the common targets were imported into the STRING database. It was then the generated PPI network was loaded into Cytoscape for the analysis seed Gene responsible for LUAD. The investigation took into account for parameters, closeness centrality, betweenness centrality, degree, and radiality. To identify network modules and SEED gene, the Molecular Complex Detection (MCODE) algorithm in Cytoscape with the following parameters: degree cutoff = 2, K-core = 2, max-depth = 100, and node score cutoff = 0.2 were used.

## Results:

In the investigation of differentially methylated regions (DMRs) in lung adenocarcinoma (LUAD), we conducted a meticulous analysis of methylation data from both cancerous and normal samples using the R package limma. Each p-value obtained from the comparison was rigorously adjusted for false discovery rate (FDR) using the Benjamini and Hochberg (BH) method, ensuring statistical robustness. DMRs were identified based on stringent criteria: an adjusted p-value < 0.05 and an absolute delta β-value > 0.2, emphasizing significant distinctions in methylation levels between LUAD and normal tissue samples. Notably, this analysis revealed a total of 7313 genes as DMRs out of the 9253 CpG sites examined. These findings highlight substantial alterations in methylation patterns across various genomic regions in LUAD, suggesting potential implications for understanding the molecular mechanisms underlying lung cancer development and progression. In the identification of differentially methylated genes (DMGs) in lung adenocarcinoma (LUAD), we employed a stringent methodology using the limma package to identify hypermethylated and hypomethylated differentially methylated regions (DMRs). Hypermethylated DMRs were identified with an adjusted p-value <0.05 and a delta β-value > 0.2, while hypomethylated DMRs were detected using a similar threshold but with a delta β-value < -0.2. This rigorous approach allowed us to pinpoint significant epigenetic alterations specific to LUAD. Additionally, we utilized the IlluminaHumanMethylationEPICanno.ilm10b2.hg19 package to match CpG loci with their corresponding genes, enabling the association of methylation changes with specific genomic regions. Among the identified genes, a total of 5,152 exhibited hypermethylation, while 2,161 genes displayed hypomethylation. These findings highlight the presence of distinct epigenetic modifications in LUAD, suggesting potential implications for understanding the underlying mechanisms of this type of lung cancer and identifying novel biomarkers or therapeutic targets.

In the identification of differentially expressed genes (DEGs) using the R-DEGSeq2 package, we ensured statistical rigor by computing adjusted p-values for each test using the Benjamini and Hochberg (BH) method to control the false discovery rate (FDR). Log-transformed expression values were then utilized to identify DEGs, specifically focusing on upregulated genes meeting the criteria of an adjusted p-value< 0.05 and a logFC (fold change) > 2. This stringent approach allowed us to pinpoint genes exhibiting substantial upregulation in expression levels. Notably, our analysis identified a total of 250 genes as upregulated out of 3212 evaluated. These findings shed light on the molecular landscape of gene expression changes associated with the condition under study, providing valuable insights into potential biomarkers or therapeutic targets. The relationship between methylation and gene expression in lung adenocarcinoma (LUAD), shows an intersection analysis of differentially methylated genes (DMGs) and differentially expressed genes (DEGs) was conducted to identify genes exhibiting hypo-methylation and upregulation respectively, referred to as hypo-Up genes. Among the 2161 hypomethylated genes (DMGs) identified, 630 were observed to be upregulated (DEGs). This intersection analysis revealed a total of 29 genes as DMEGs, indicating simultaneous changes in methylation and expression levels associated with LUAD. The identified genes include COL1A1, PHLDA2, PPP1R1B, FA2H, SOX9, WNT7B, ATN1, GPRIN1, NUDT8, NPM3, CERCAM, CDH1, LOXL1, AP1S1, TIMELESS, LAD1, WDR18, MICALL2, SLC34A2, MANSC1, CMTM8, EXOSC4, SLC27A4, FAM50A, MRPL3, TMEM97, PACS1, SNAPIN, KRCC1.These findings provide insights into potential regulatory mechanisms underlying LUAD pathogenesis and may serve as valuable candidates for further investigation as biomarkers or therapeutic targets. The obtained 29 potential Common DM-DE targets were transferred to STRING platform (https://string-db.org/) for PPI analysis and visualized Cytoscape Software for visualizing and MCODE tool in cytoscape to analysing Seed gene. There show three clusters and from the three COL1A1 was identified as the seed gene with MCODE Score of 2.0.

## Discussion:

The integrative multi-omics strategy used in this work successfully identified numerous possible biomarkers in lung adenocarcinoma (LUAD) by combining DNA methylation and gene expression profiles. By focusing on hypomethylated and upregulated genes, the study emphasizes the importance of epigenetic dysregulation in LUAD progression. The significance of DNA methylation alterations in cancer has been well acknowledged since the early 2000s. Esteller found that abnormal DNA methylation patterns, such as hypermethylation and global hypomethylation, play critical roles in carcinogenesis by silencing tumor suppressor genes and activating oncogenes [3]. Our data confirm this idea by showing that several hypomethylated genes have increased expression levels in LUAD, implying that methylation loss leads to gene activation and may accelerate neoplastic processes. Furthermore, Baylin and Herman (2000) stressed that genetic and epigenetic changes interact to induce cancer progression [9]. This dual process is obvious in LUAD, where our research revealed extensive methylation changes in conjunction with large gene expression changes. Such integrated analysis enables the identification of genes whose dysregulation may not be detectable using single-omics techniques, consistent with the multi-layered perspective advocated in early cancer epigenetic research.

The analytical methodology used in this work is consistent with prior methodologies, such as those described by Wang *et al.* 2020, who found that combining DNA methylation and gene expression data improves biomarker discovery in lung cancer [1]. This integrative technique not only improves the finding of biologically relevant targets, but it also helps to understand the regulatory mechanisms underlying LUAD. The identification of differentially methylated and differentially expressed genes (DMEGs) using rigorous computational analysis increases the biomarkers' potential therapeutic value. Early investigations by Esteller *et al.* (2001) stressed the relevance of genome-wide methylation profiling in identifying cancer-associated epigenetic markers [10], a strategy that has been effectively applied to LUAD. The study's use of expression profiling adds to the body of evidence showing epigenetic instability is frequently associated to transcriptional activation or repression in cancer cells. Although this study focuses on biomarker identification, future research may look at therapeutic implications, such as drug repositioning and target validation. Previous research has shown that combining computational techniques with molecular docking can help find and assess novel medication candidates for cancer therapy [5]. Building on these approaches may increase the translational potential of the identified biomarkers.

## Conclusion:

Integrating DNA methylation and gene expression data provides a powerful approach for identifying novel biomarkers in lung adenocarcinoma (LUAD), highlighting the role of epigenetic deregulation and uncovering genes potentially crucial for cancer progression and therapy resistance. These candidate biomarkers offer promising avenues for improved early detection, prognosis, and the development of precision medicine strategies tailored to the molecular profile of individual LUAD tumors. Further experimental and clinical validation is needed to confirm the functional significance and clinical utility of these biomarkers, paving the way for future advances in LUAD research and cancer epigenomics.
